# Event-Triggered Robust State Estimation for Nonlinear Networked Systems with Measurement Delays against DoS Attacks

**DOI:** 10.3390/s23146553

**Published:** 2023-07-20

**Authors:** Min Wang, Huabo Liu

**Affiliations:** 1 School of Automation, Qingdao University, Qingdao 266071, China; 2021023684@qdu.edu.cn; 2 Shandong Key Laboratory of Industrial Control Technology, Qingdao 266071, China

**Keywords:** nonlinear systems, robust state estimation, linearization errors, denial-of-service attacks, measurement delay, event-triggered mechanism

## Abstract

In this paper, we focus on the event-triggered robust state estimation problems for nonlinear networked systems with constant measurement delays against denial-of-service (DoS) attacks. The computation of the extended Kalman filter (EKF) generates errors of linearization approximations, which can result in increased state estimation errors, and subsequently amplifies the linearization errors. DoS attacks interfere with the transmission of measurements sent to the remote robust state estimator by overloading the communication networks, while the communication rate of the communication channel is constrained. Therefore, an event-triggered robust state estimation algorithm based on sensitivity penalization with an explicit packet arrival parameter is derived to defend against DoS attacks and linearization errors. Meanwhile, the presence of measurement delays precludes the direct use of conventional state estimation algorithms, prompting us to devise an innovative state augmentation method. The results of the numerical simulations show that the proposed robust state estimator can appreciably improve the accuracy of state estimation.

## 1. Introduction

Networked systems have achieved successful applications in various fields, including civil infrastructure, environmental monitoring, intelligent transportation, and smart grids [1]. The performance of various components within a networked system is contingent upon the accuracy of data transmission over the communication network, which is determined by the capabilities of the measurement and data transmission technologies employed. Recent advancements have shown a proclivity toward wireless shared communication networks over wired or dedicated networks [2]. Although the adoption of multi-purpose communication networks can reduce maintenance costs and provide flexibility in system architecture design, it also introduces new challenges. Open wireless communication networks, in particular, are vulnerable to network attacks and delays as opposed to separate dedicated network communication channels [2,3]. For this reason, an increasing number of studies focus on the security of wireless networked systems.

Deception attacks, denial-of-service (DoS) attacks, and replay attacks are among the most prevalent forms of attack in networked systems [4,5,6]. Due to the ease of implementation, DoS attacks have become one of the most common threats to networked systems. These attacks interfere with communication channels between sensors and robust state estimators, disrupting the availability of resources and resulting in packet dropping [7]. Various results have been published for the security of networked systems under DoS attacks. The measurement of missing phenomena caused by DoS attacks can be modeled using two fundamental methods: a binary switching sequence or a Markov chain. The security control of networked switched systems under the threat of DoS attacks during the transmission of output information from sensors to controllers is investigated in [8]. By assuming that DoS attacks follow a Bernoulli distribution, an event-triggered sampling mechanism is proposed to reduce the communication burden while preserving satisfactory performance. In [9], two independent Bernoulli distributions are used to model periodic DoS attacks and random packet dropout for cyber-physical systems. In [10], an aperiodic DoS attack is introduced, characterized by its duration and frequency, and a switched fuzzy Markov jump closed-loop system is established. An event-triggered scheme that incorporates membership functions is proposed to address the mismatched behavior between the membership functions of a fuzzy system and its fuzzy controller. The problem of event-triggered resilient $L_{\infty }$ control for the Markov closed-loop jump system subject to DoS attacks is considered in [11], where criteria are proposed to ensure the $L_{\infty }$ control performance of the system.

In the literature mentioned above, network communication is considered to be continuous, leading to a waste of communication resources. By reducing unnecessary data transmissions, an event-triggered mechanism helps reduce network traffic or congestion and mitigate network-related phenomena such as transmission delays and packet loss, thereby satisfying the basic quality-of-service requirements in the design of networked system estimators. An event-triggered mechanism can achieve a balance between the desired system estimation performance and resource utilization efficiency. In [12], a dynamic event-triggered strategy is proposed, which offers greater flexibility in setting the event-trigger threshold. The application of a node-based adaptive strategy eliminates the need for global information in the investigation of consensus for multi-agent systems. In [13], a new dynamic event-triggered mechanism is introduced for event-triggered control systems, featuring the inclusion of an internal dynamic variable. A general framework for the event-triggered stabilization of nonlinear systems using hybrid system tools is presented in [14]. This framework encompasses a wide range of existing event-triggered control techniques and provides a basis for their generalization and revision. The use of deterministic event triggers to schedule sensors destroys the Gaussian property of the state, rendering it computationally intractable to obtain an exact minimum mean-squared-error estimate. In response, a stochastic event-triggered sensor schedule for state estimation is introduced in [15], which is designed to preserve the Gaussianity of the system. Time delays are a common occurrence in complex networks due to factors such as network traffic congestion and the finite speed of signal transmission over links. The preponderance of the existing literature on the control and state estimation of systems with delays pertains to delays in inputs or actuators. Nonetheless, in networked systems, delays can also originate from sensor measurements or communication networks. The delay of most sensors and communication networks is similar or slightly modified due to their acquisition environment, acquisition algorithm, and transmission environment. Considering that subtle changes have less impact in the discrete domain, it is reasonable to assume that the measurement delay is constant. The decision-making process of a controller within a networked system depends on data from sensor measurements, and any delay in measurements or network transmission can adversely affect the system’s performance. Therefore, it is crucial to have an accurate real-time estimator capable of providing an accurate state of the system in the presence of delays.

Measurement delay in state estimation is a well-known issue, often referred to as the time-varying measurement problem, the time-delayed measurement problem, or the out-of-sequence measurement (OOSM) problem. Four distinct approaches exist for addressing problems with known delays: prediction, extrapolation, re-organized innovation, and state augmentation. Prediction, when applied within the Kalman filter algorithm, provides a solution to the one-step delayed OOSM problem. In [16], a method for forward prediction of OOSMs that does not rely on retrodiction is proposed. The tracklet is predicted forward and de-correlated from the actual track using a track de-correlation method similar to the information filter approach before being fused with the actual track. This method, referred to as forward-prediction fusion and de-correlation, has been shown to compare favorably to retrodiction-based algorithms while requiring less data storage in most cases. The extrapolation approach is a second viable method for addressing time-delayed measurement problems. In [17], a method involving the “extrapolation” of measurements to the current moment using past and present estimates of the Kalman filter is proposed, and the optimal gain of the extrapolated measurements is determined. Re-organized innovation is derived based on the projection in Hilbert space and an innovation analysis method. In [18], a system with *l*-time delayed measurements is studied using re-organized innovation analysis. State augmentation is an effective approach for tackling the time-delayed measurement problem. The approach involves utilizing the delayed measurement to estimate the state of the corresponding past moment and deriving the current state prediction from this corrected past state. The key to this approach lies in augmenting the state vector in a skillful manner and establishing a correlation between the augmented state vector, which includes the corresponding past state, and the delayed measurement. An augmented state Kalman filter is proposed in [19] to address the state estimation problem with time-delayed measurements. The uncertainty of the delay time is resolved based on its probability distribution. In [20], the problem of state estimation in the presence of delay uncertainty is studied and uncertain delay is represented as a probability density function. The proposed estimator addresses the impact of uncertain delayed measurements by incorporating an augmented state Kalman filter.

State estimation, commonly employed in automatic control and signal processing, refers to the process of estimating the internal state of a dynamic system based on available measurement data. Many conditions in the dynamic and measurement processes of real networked systems are nonlinear [21,22]. As a result, various estimation methods for nonlinear systems have been proposed, including the extended Kalman filter (EKF) [23], unscented Kalman filter (UKF) [24], cubature Kalman filter [25], and so on. Ref. [26] presents a method for nonlinear state estimation of biomass in a batch bioprocess, which employs the EKF with a sample-state augmentation method to incorporate delayed measurements. A dynamic state estimation algorithm is proposed in [27], which employs Holt’s two-parameter exponential smoothing and extended Kalman filtering techniques. The recursion formula for parameter identification, state prediction, and state filtering incorporates the statistical characteristics of data packet losses caused by DoS attacks. However, all these techniques assume that the system model is accurate and do not account for linearization errors.

The EKF is an efficient method for state estimation of a nonlinear state-space model, which is an expanded version of the standard Kalman filter. However, this approach may suffer performance degradation if there are considerable linearization errors caused by first-order linear approximations. To enhance estimation performance, various methods have been developed to address linearization errors. In [28], an adaptive loop is proposed that repeatedly executes a nonlinear solver on a fixed mesh until the linearization error estimate falls below the discretization error estimate. The mesh is then adaptively refined and the loop continues. In order to overcome the limitations imposed by linearization, the unscented transformation is introduced in [29] as a method for conveying mean and covariance information through nonlinear transformations. It is more precise, simpler to implement, and requires the same order of calculations as linearization. A robust tracking technique for the heating value in an underground coal gasification process is presented in [30], which utilizes dynamic integral sliding-mode control and a gain-scheduled modified Utkin observer. This control scheme can effectively handle parametric uncertainties, measurement noise, and water influx disturbance. A robust EKF is developed in [31] to provide an optimized upper bound on the state estimation error covariance, even in the face of model uncertainties and linearization errors. It possesses robustness against process noises, measurement noises, linearization errors, and model uncertainties.

Although there is extensive research on nonlinear systems, as indicated by the above analysis, few papers in the public domain specifically address methods for simultaneously overcoming constant measurement delays, DoS attacks, and linearization errors in nonlinear systems. In this paper, we concentrate on the event-triggered robust state estimation for nonlinear networked systems with constant measurement delays against DoS attacks. The robust state estimation algorithm is investigated based on the connection between the Kalman filter and the regularized least-squares problem. To reduce the transmission burden on the communication network, an event-triggered mechanism is implemented in the estimation process and a binary variable is used to represent the packet-sending parameter. Packet loss resulting from DoS attacks that jam communication networks is characterized by a Bernoulli distribution. Subsequently, a packet-arrival parameter that contains information about both the packet-sending and packet-loss parameters is explicitly included in the improved cost function. We meticulously design a specific state augmentation method to address constant measurement delays and modify the cost function of the regularized least-squares problem to account for linearization errors. An analytic expression for the robust state estimator is obtained, which is recursively implementable and has a form similar to the EKF. Furthermore, we design numerical simulations to confirm the efficacy of the proposed event-triggered robust state estimator.

The remainder of this paper is organized as follows. In Section 2, we present our model of packet loss due to DoS attacks and the packet transmission of the event trigger, as well as introduce an event-triggered nonlinear system model with a constant measurement delay under DoS attack conditions. In Section 3, a time-delay model is transformed into a formally non-time-delay model using a state augmentation method, an improved event-triggered model with a fixed measurement delay under DoS attacks is obtained, and a robust state estimation algorithm that employs a sensitivity penalty for the nonlinear networked system is proposed. In Section 4, we present numerical examples that demonstrate the efficacy of the estimator proposed in this paper. Finally, Section 5 summarizes the work of this paper.

Notations: N denotes the set of natural numbers, including zero. $R^{n}$ denotes the n-dimensional Euclidean space. ${∥x∥}_{V}$ denotes the norm with weighted coefficients $\sqrt{x^{T}Vx}$. The Euclidean norm for real vectors is denoted by $∥*∥$. E(∗) represents the expectation of a random vector or matrix and Pr(∗) represents the probability of *. var(∗) denotes the variance of *. $col\left\{*\right\}$ indicates the operation bracket of the stacking vector or matrix. $diag\left\{*\right\}$ is a block diagonal matrix.

## 2. Problem Formulation

**Figure 1:**
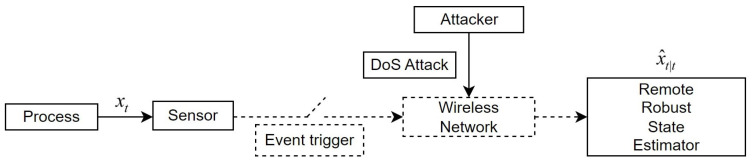
Framework of the event-triggered nonlinear networked system against DoS attacks.

**Figure 2:**
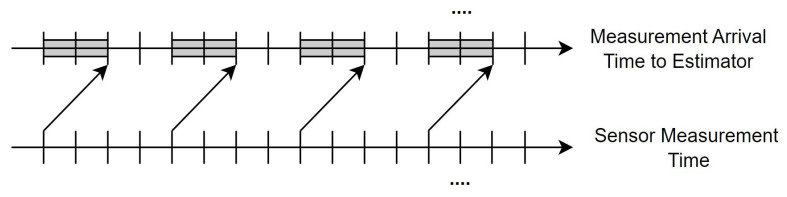
Measurement data transmission with fixed delay.

We present a nonlinear networked system structure for even-triggered remote robust state estimation with a measurement delay against DoS attacks, as depicted in Figure 1. It consists of six main parts: a process, a sensor, an event trigger, a wireless network, an attacker, and a remote robust state estimator. The sensor continuously sends measurement values to the event trigger, where the event-triggered transmission mechanism decides if transmission to the remote state estimator over the wireless communication network is necessary. In addition to the packet loss caused by DoS attacks from network attackers, the wireless communication network also experiences transmission delays due to its structure, as shown in Figure 2.

**Figure 3:**
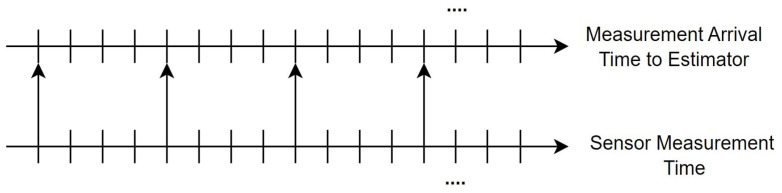
Ideal measurement data transmission situation.

If a sensor takes a measurement at the same instant that the measurement value is available in a filter, a general filtering algorithm such as the EKF can generate a consistent and correct estimation result. This ideal scenario is represented in Figure 3, where there is no time delay in the system. In contrast, in a delayed networked system, the time at which the sensor sends out the measurement value and the time at which it is received by the remote state estimator do not coincide, as shown in Figure 2.

An event-triggered transmission strategy is an effective method for reducing communication rates and alleviating bandwidth pressure on wireless communication networks. We consider a noisy measurement channel

(1) $$\begin{array}{c} y_{t}=h\left(x_{t}\right)+v_{t}, \end{array}$$

where $x_{t}\in R^{n}$ and $y_{t}\in R^{m}$ denote the state vector and measurement output, respectively. $v_{t}\in R^{m}$ represents the effect of communication noise. $h\left(\cdot \right):R^{n}\to R^{m}$ is a nonlinear function and is assumed to be continuously differentiable. Let $c_{t}$ represent a packet-sending parameter, which is a binary variable taking the value of 1 if the data packet $y_{t}$ is transmitted at time instant *t*, and 0 otherwise. This relationship can be expressed as follows:

(2) $$\begin{array}{c} c_{t}=\left\{\begin{array}{cc} 0, & \;\mathrm{if}\;{∥y_{t}-{\tilde{y}}_{t}∥}^{2}\leq \Xi ,\\ 1, & \;\mathrm{otherwise}, \end{array}\right. \end{array}$$

where the vectors ${\tilde{y}}_{t}=y_{t-n_{t}}\in R^{m}$. $n_{t}\geq 0$ denotes the number of time instants elapsed since the last transmission of the sensor, i.e., $n_{t}$ is such that $c_{t-n_{t}}=1$ and $c_{t-1}=\cdot \cdot \cdot =c_{t-n_{t}+1}=0$. The positive real Ξ has to be chosen to ensure that it satisfies the following transmission rate constraint:

(3) $$\begin{array}{c} \lim_{\tau \to \infty }\frac{1}{\tau }\sum_{t=1}^{\tau }E\left\{c_{t}\right\}=\alpha , \end{array}$$

where the transmission rate α∈(0,1). For any given desired transmission rate α, the threshold Ξ can be readily determined by $\Xi =\epsilon_{m}^{-1}\left(1-\alpha \right)$, where $\epsilon_{m}\left(\cdot \right)$ is the cumulative distribution function of an $\chi^{2}$ random variable with *m* degrees of freedom.

In a wireless networked system, the inherent openness of the wireless communication network makes it vulnerable to malicious network attacks such as DoS attacks—one of the most common and typical types of network attacks. When a DoS attack occurs, network congestion can lead to the loss or even continuous loss of sensor measurement data packets. This packet loss is time-varying due to the limited power of DoS attacks, which affects the state estimation results and endangers the safe operation of the system. Let $\gamma_{t}$ represent a packet-dropping parameter, which is determined depending on whether the communication channel is congested by an attacker using DoS attack techniques. We assume that the sequence of $\gamma_{t}$ is independent and identically distributed, forming a Bernoulli process, which is used to illustrate the stochastic property of packet sequential loss. Therefore, the packet-dropping parameter $\gamma_{t}$ can be expressed as follows:

$$\mathrm{Pr}\left(\gamma_{t}=1\right)=1-\rho ,\mathrm{Pr}\left(\gamma_{t}=0\right)=\rho ,var\left(\gamma_{t}\right)=\rho \left(1-\rho \right),E\left(\gamma_{t}\right)=1-\rho .$$

$\gamma_{t}$ predicts the occurrence of packet loss caused by DoS attacks at time instant *t*, which receives a value of 1 when the data packet is successfully delivered and a value of 0 when the communication channel is congested due to DoS attacks. The packet-dropping rate is represented by $\rho \in \left(0,1\right)$.

For the convenience of representation and calculation, let $\psi_{t}=c_{t}\gamma_{t}$ represent a packet-arrival parameter, which can be expressed as follows:

(4) $$\begin{array}{c} \psi_{t}=\left\{\begin{array}{cc} 1, & \;\mathrm{if}\;c_{t}=1,\gamma_{t}=1,\mathrm{sent}\;\mathrm{and}\;\mathrm{transmitted}\;\mathrm{normally},\\ 0, & \;\mathrm{if}\;c_{t}=1,\gamma_{t}=0,\mathrm{sent}\;\mathrm{but}\;\mathrm{transmission}\;\mathrm{failed},\\ 0, & \;\mathrm{if}\;c_{t}=0,\mathrm{unsent}. \end{array}\right. \end{array}$$

This parameter represents whether the remote state estimator has received the packet from the event trigger. At time instant *t* when the remote robust state estimator successfully receives the packet, $\psi_{t}=1$; otherwise, $\psi_{t}=0$. In practical engineering, the packet-arrival parameter $\psi_{t}$ can be achieved through timestamp technology.

Consider an event-triggered nonlinear networked system $\Sigma_{1}$ with a constant measurement delay under DoS attacks. The plant dynamics and its output measurements received by a remote robust state estimator are assumed to be describable using the following discrete model:

Σ1:{(5)xt+1=fxt,ut+wt(6)yt=ψthxt−d+vt,t≥0,

where t∈N represents the time index. The variable d∈N indicates the number of time-delayed frames of the measurement signal, which is known and time-invariant. $u_{t}\in R^{l}$ is a known external input signal. The vector $w_{t}\in R^{n}$ denotes the process noises. $f\left(\cdot ,\cdot \right):R^{n}\times R^{l}\to R^{n}$, which is nonlinear, is assumed to possess continuous differentiability. It is assumed that $x_{t}$, $w_{t}$, and $v_{t}$ are uncorrelated random vectors, with $E(col\left\{w_{t},v_{t},x_{0}\right\})=0$ and $E\left(col\left\{x_{0},w_{t},v_{t}\right\}col^{T}\left\{x_{0},w_{s},v_{s}\right\}\right)=diag\left\{P_{0},Q_{t}\delta_{ts},R_{t}\delta_{ts}\right\}$,∀t,s>0, where $P_{0}$, $Q_{t}$, and $R_{t}$ are known positive definite matrices, and $\delta_{ts}$ represents the Kronecker delta function, which equals 1 when t=s and 0 whenever t≠s. $\psi_{t}$ is the packet-arrival parameter, which is equal to 1 or 0, and needs to be adjusted according to whether the data packet arrives or not.

## 3. Design of the Robust State Estimator

**Figure 4:**
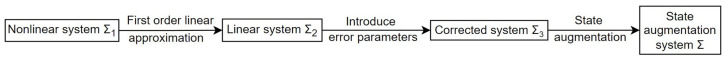
The flow diagram of the system processing.

We perform the three-step operation shown in Figure 4 on nonlinear system $\Sigma_{1}$ to further design the robust state estimation algorithm.

In the same manner as with the EKF, we first perform a first-order Taylor approximation to the nonlinear system $\Sigma_{1}$. By linearizing the nonlinear function f(·,·) in Equation (5) and the function h(·) in Equation (6) at the posterior estimate and the prior estimate, respectively, and omitting higher-order infinitesimals, we can approximate the nonlinear system as follows:

Σ2:{(7)xt+1=f(x^t∣t,ut)+At(x^t∣t)(xt−x^t∣t)+wt(8)yt=ψt(h(x^t−d∣t−d−1)+Ct−d(x^t−d∣t−d−1)(xt−d−x^t−d∣t−d−1))+vt,t≥0.

The matrices in the above equation are defined as

(9) At(x^t∣t)=∂f(x,u)∂x|x=x^t∣tu=ut,Ct−d(x^t−d∣t−d−1)=∂h(x)∂x|x=x^t−d∣t−d−1,

which are known Jacobian matrices at the *t*-th sampled instant. We abbreviate $A_{t}\left({\hat{x}}_{t|t}\right)$ and $C_{t-d}\left({\hat{x}}_{t-d|t-d-1}\right)$ to $A_{t}$ and $C_{t}$, respectively. ${\hat{x}}_{t|k}$ represents the state estimate based on the measurement output $y_{t}|{}_{t=0}^{k}$.

Linearization errors can severely degrade the performance of the EKF, particularly when heavily weighted. To mitigate these problems, it is necessary to implement specific considerations and employ an estimation algorithm that is robust to linearization errors. By combining the known terms of the system $\Sigma_{2}$ and taking into account linearization errors in state estimations, we revise the system $\Sigma_{2}$ as the following system:

Σ3:{(10)xt+1=At(x^t∣t,εt1)xt+at+wt(11)yt=ψtCt−d(x^t−d∣t−d−1,εt−d2)xt−d+bt−d+vt,t≥0,

where $a_{t}=f\left({\hat{x}}_{t|t},u_{t}\right)-A_{t}\left({\hat{x}}_{t|t}\right){\hat{x}}_{t|t}$ and $b_{t-d}=h\left({\hat{x}}_{t-d|t-d-1}\right)-C_{t-d}\left({\hat{x}}_{t-d|t-d-1}\right){\hat{x}}_{t-d|t-d-1}$, which are known values at the *t*-th sampled instant. We abbreviate $A_{t}\left({\hat{x}}_{t|t},\varepsilon_{t}\right)$ and $C_{t}\left({\hat{x}}_{t|t-1},\varepsilon_{t}\right)$ to $A_{t}\left(\varepsilon_{t}\right)$ and $C_{t}\left(\varepsilon_{t}\right)$, respectively. In addition, $\varepsilon_{t}^{1}$ and $\varepsilon_{t}^{2}$ denote the differences between the true value of the plant state vector and its posterior and prior estimates at time t, respectively. To be more precise, $A_{t}\left({\hat{x}}_{t|t},\varepsilon_{t}^{1}\right)$ is composed of ${\hat{x}}_{t|t}+\varepsilon_{t}^{1}$ and $C_{t}\left({\hat{x}}_{t|t-1},\varepsilon_{t}^{2}\right)$ is composed of ${\hat{x}}_{t|t-1}+\varepsilon_{t}^{2}$, which corresponds to the true value of the plant state vector.

In order to make the corresponding estimation problem mathematically tractable, the elements of the state estimation errors, namely $\varepsilon_{t,k}^{1}$ and $\varepsilon_{t,k}^{2}$, k=1,2…,n, are assumed to be independent of each other. Specifically, the matrices $A_{t}\left({\hat{x}}_{t|t},\varepsilon_{t}^{1}\right)$ and $C_{t}\left({\hat{x}}_{t|t-1},\varepsilon_{t}^{2}\right)$ are defined in the same manner as $A_{t}\left({\hat{x}}_{t|t}\right)$ and $C_{t}\left({\hat{x}}_{t|t-1}\right)$ in Equation (9). In other words, the definitions of these matrices are identical to those of $A_{t}\left({\hat{x}}_{t|t}\right)$ and $C_{t}\left({\hat{x}}_{t|t-1}\right)$, respectively.

(12) At(x^t∣t,εt1)=∂f(x,u)∂x|x=x^t∣t+εt1u=ut,Ct−d(x^t−d∣t−d−1,εt−d2)=∂h(x)∂x|x=x^t−d∣t−d−1+εt−d2.

By expanding $A_{t}\left({\hat{x}}_{t|t},\varepsilon_{t}^{1}\right)$ at $\varepsilon_{t}^{1}=0$, we obtain

(13) $$A_{t}\left({\hat{x}}_{t|t},\varepsilon_{t}^{1}\right)=A_{t}\left({\hat{x}}_{t|t}\right)+\sum_{j}^{n}\Delta A_{t,j}\varepsilon_{t,j}^{1}+o\left({∥\varepsilon_{t}^{1}∥}_{2}^{2}\right),$$

where $\varepsilon_{t,j}^{1}$ denotes the *j*-th component of $\varepsilon_{t}^{1}$, $\Delta A_{t,j}=\frac{\partial A_{t}\left({\hat{x}}_{t|t},\varepsilon_{t}\right)}{\partial \varepsilon_{i,j}}|{}_{\varepsilon_{i,j}=0}$. Furthermore, we obtain

(14) $$A_{t}\left({\hat{x}}_{t|t},\varepsilon_{t}^{1}\right)=A_{t}\left({\hat{x}}_{t|t}\right)+{\left(\varepsilon_{t}^{1}⊗I\right)}^{T}\Delta A_{t}+o\left({∥\varepsilon_{t}^{1}∥}_{2}^{2}\right),$$

where $\Delta A_{t}=col\left(\Delta A_{t,j}\right)$. The system matrices $A_{t}\left({\hat{x}}_{t|t},\varepsilon_{t}^{1}\right)$ and $C_{t}\left({\hat{x}}_{t|t-1},\varepsilon_{t}^{2}\right)$ are differentiable at every time instant with respect to every element of $\varepsilon_{t}^{1}$ and $\varepsilon_{t}^{2}$, respectively.

Since conventional state estimators such as the EKF are not directly applicable to systems with time delays, we implement state augmentation to transform the measurement-delay system $\Sigma_{3}$ into a system without time delays in its form.

**Theorem 1.**  *Under the assumption that the number of measurement-delay frames is known and constant, we construct the augmented state $X_{t}=\mathrm{col}\left[x_{t}\;\Delta_{t}\;\Delta_{t-1}\cdots \Delta_{t-d+1}\right]$, where $\Delta_{t}=C_{t-1}\left(\varepsilon_{t-1}^{2}\right)x_{t-1}+b_{t-1}-C_{t}\left(\varepsilon_{t}^{1}\right)x_{t}-b_{t}$. We transform the system $\Sigma_{3}$ into the following equivalent model: *

Σ:{(15)Xt+1=A˜tεt1,εt+12Xt+Dtdt+B˜tεt+12at+B˜tεt+12wt,(16)yt=ψtC˜tεt1Xt+bt+vt,t≥0,

*where*

(17) $$\begin{array}{c} D_{t}=\left[\begin{array}{c} 0_{n\times m}\\ I_{m}\\ 0_{(d-1)m\times m} \end{array}\right], \end{array}$$

(18) $$\begin{array}{c} \;d_{t}=b_{t}-b_{t+1}, \end{array}$$

(19) $$\begin{array}{cc} {\tilde{A}}_{t}\left(\varepsilon_{t}^{1},\varepsilon_{t+1}^{2}\right) & =\left[\begin{array}{ccc} A_{t}\left(\varepsilon_{t}^{1}\right) & 0_{n\times (d-1)m} & 0_{n\times m}\\ C_{t}\left(\varepsilon_{t}^{1}\right)-C_{t+1}\left(\varepsilon_{t+1}^{2}\right)A_{t}\left(\varepsilon_{t}^{1}\right) & 0_{m\times (d-1)m} & 0_{m\times m}\\ 0_{(d-1)m\times n} & I_{(d-1)m} & 0_{(d-1)m\times m} \end{array}\right], \end{array}$$

(20) $$\begin{array}{cc} {\tilde{B}}_{t}\left(\varepsilon_{t+1}^{2}\right) & =\left[\begin{array}{c} I_{n\times n}\\ -C_{t+1}\left(\varepsilon_{t+1}^{2}\right)\\ 0_{(d-1)m\times n} \end{array}\right], \end{array}$$

(21) $$\begin{array}{cc} {\tilde{C}}_{t}\left(\varepsilon_{t}^{1}\right) & =\left[C_{t}\left(\varepsilon_{t}^{1}\right)\;\overset{d}{\overbrace{I_{m}\cdots I_{m}}}\right]. \end{array}$$

**Proof of Theorem 1.**  First, we define

(22) $$z_{t}\overset{\Delta }{\;=}C_{t-d}\left({\hat{x}}_{t-d|t-d-1},\varepsilon_{t-d}^{2}\right)x_{t-d}+b_{t-d}-C_{t}\left({\hat{x}}_{t|t-1},\varepsilon_{t}^{2}\right)x_{t}-b_{t}.$$

Thus, the system $\Sigma_{2}$ can be re-expressed as the following system:

{(23)xt+1=At(x^t∣t,εt1)xt+at+wt(24)yt=ψtCt(x^t∣t−1,εt2)xt+bt+zt+vt,t≥0,

By combining ${\tilde{A}}_{t}\left(\varepsilon_{t}^{1},\varepsilon_{t+1}^{2}\right)$ and $X_{t}$, and calculating ${\tilde{A}}_{t}\left(\varepsilon_{t}^{1},\varepsilon_{t+1}^{2}\right)X_{t}$, we can obtain

$$\begin{array}{cc} {\tilde{A}}_{t}\left(\varepsilon_{t}^{1},\varepsilon_{t+1}^{2}\right)X_{t} & =\left[\begin{array}{ccc} A_{t}\left(\varepsilon_{t}^{1}\right) & 0_{n\times (d-1)m} & 0_{n\times m}\\ C_{t}\left(\varepsilon_{t}^{1}\right)-C_{t+1}\left(\varepsilon_{t+1}^{2}\right)A_{t}\left(\varepsilon_{t}^{1}\right) & 0_{m\times (d-1)m} & 0_{m\times m}\\ 0_{(d-1)m\times n} & I_{(d-1)m} & 0_{(d-1)m\times m} \end{array}\right] \end{array}$$

(25) $$\begin{array}{cc} \times \left[\begin{array}{c} x_{t}\\ \Delta_{t}\\ \Delta_{t-1}\\ \vdots \\ \Delta_{t-d+1} \end{array}\right]\; & =\left[\begin{array}{c} A_{t}\left(\varepsilon_{t}^{1}\right)x_{t}\\ C_{t}\left(\varepsilon_{t}^{1}\right)x_{t}-C_{t+1}\left(\varepsilon_{t+1}^{2}\right)A_{t}\left(\varepsilon_{t}^{1}\right)x_{t}\\ \Delta_{t}\\ \vdots \\ \Delta_{t-d+2} \end{array}\right]. \end{array}$$

Combining Equations (17)–(20) and (25) yields

(26) $$\begin{array}{cc} \; & {\tilde{A}}_{t}\left(\varepsilon_{t}^{1},\varepsilon_{t+1}^{2}\right)X_{t}+D_{t}d_{t}+{\tilde{B}}_{t}\left(\varepsilon_{t+1}^{2}\right)a_{t}+{\tilde{B}}_{t}\left(\varepsilon_{t+1}^{2}\right)w_{t}\\ \; & =\left[\begin{array}{c} A_{t}\left(\varepsilon_{t}^{1}\right)x_{t}+a_{t}+w_{t}\\ C_{t}\left(\varepsilon_{t}^{1}\right)x_{t}+b_{t}-C_{t+1}\left(\varepsilon_{t+1}^{2}\right)\left[A_{t}\left(\varepsilon_{t}^{1}\right)x_{t}+a_{t}+w_{t}\right]-b_{t+1}\\ \Delta_{t}\\ \vdots \\ \Delta_{t-d+2} \end{array}\right]=\left[\begin{array}{c} x_{t+1}\\ \Delta_{t+1}\\ \Delta_{t}\\ \vdots \\ \Delta_{t-d+2} \end{array}\right]=X_{t+1}. \end{array}$$

From (21), we can deduce that

(27) $$\begin{array}{cc} \; & \psi_{t}\left({\tilde{C}}_{t}\left(\varepsilon_{t}^{1}\right)X_{t}+b_{t}\right)+v_{t}=\psi_{t}\left(C_{t}\left(\varepsilon_{t}^{1}\right)x_{t}+b_{t}+\Delta_{t}+\cdots +\Delta_{t-d+1}\right)+v_{t}\\ \; & =\psi_{t}\left[C_{t-d}\left(\varepsilon_{t-d}^{2}\right)x_{t-d}+b_{t-d}\right]+v_{t}=y_{t}. \end{array}$$

By combining (26) and (27), we can obtain the system Σ. The proof of Theorem 1 ends here. □

State augmentation is a straightforward yet potent method that entails modifications to the parameter matrix. This transformation results in the system Σ with no measurement delay in its form and an increase in the system dimension from *n* to n+md. The proposed state augmentation method indirectly establishes a correlation between the delayed measurements and the current state. As the time delay becomes larger, the system requires more computational resources to process and handle the delay. More specifically, the computational burden of the system does indeed increase correspondingly with the increase in the measurement delay. However, in practical engineering applications, the degree of the time delay is typically not excessive, minimizing the impact of this issue. During the derivation of the state estimator, the number of time-delayed frames, denoted as *d*, is required as it is included in both the coefficient matrix and the augmented state $X_{t}$.

According to [32], the Kalman filter can be interpreted as the solution of a regularized least-squares (RLS) problem, which for the system Σ can be expressed as

(28) $$\begin{array}{c} {\hat{X}}_{t+1|t+1}={\tilde{A}}_{t}\left(0,0\right){\hat{X}}_{t|t+1}+D_{t}d_{t}+{\tilde{B}}_{t}\left(0\right)a_{t}+{\tilde{B}}_{t}\left(0\right){\hat{w}}_{t|t+1},\\ \left(\begin{array}{c} {\hat{X}}_{t|t+1}\\ {\hat{w}}_{t|t+1} \end{array}\right)=\arg\min_{X_{t|t+1},w_{t|t+1}}\left[{∥X_{t|t+1}-{\hat{X}}_{t|t}∥}_{P_{t|t}^{-1}}^{2}+{∥w_{t|t+1}∥}_{Q_{t}^{-1}}^{2}+{∥e_{t}\left(0,0\right)∥}_{R_{t+1}^{-1}}^{2}\right], \end{array}$$

where $e_{t}\left(0,0\right)=y_{t+1}-b_{t+1}-{\tilde{C}}_{t+1}\left(0\right)\left[{\tilde{A}}_{t}\left(0,0\right)X_{t|t+1}+D_{t}d_{t}+{\tilde{B}}_{t}\left(0\right)a_{t}+{\tilde{B}}_{t}\left(0\right)w_{t|t+1}\right]$. The objective of the RLS problem is to ameliorate the estimation through the inclusion of new measurements; however, its effectiveness may be constrained by linearization errors.

Considering the appreciable deterioration of estimation performance because of linearization errors, which are generally unavoidable, and the pockets of sensor measurement data that do not arrive due to malicious network attacks and event triggers, we improve the cost function of the RLS problem as follows:

(29) JXt|t+1,wt|t+1=12μtXt|t+1−X^t∣tPt∣t−12+wt|t+1Qt−12+ψt+1μtet(0,0)Rt+1−12+1−μt∑k=1n∂et(εt1,εt+12)∂εt,k122+∂et(εt1,εt+12)∂εt+1,k222εt1=0εt+12=0,

where $e_{t}\left(\varepsilon_{t}^{1},\varepsilon_{t+1}^{2}\right)=y_{t+1}-b_{t+1}-{\tilde{C}}_{t+1}\left(\varepsilon_{t+1}^{2}\right)\left[{\tilde{A}}_{t}\left(\varepsilon_{t}^{1},\varepsilon_{t+1}^{2}\right)X_{t|t+1}+D_{t}d_{t}+{\tilde{B}}_{t}\left(\varepsilon_{t+1}^{2}\right)a_{t}+{\tilde{B}}_{t}\left(\varepsilon_{t+1}^{2}\right)w_{t|t+1}\right]$. When disregarding the linearization error, we can obtain the cost function of the RLS problem, which is Equation (28). However, a large linearization error can reduce the estimation accuracy of the estimator and even cause a divergence of the estimation values. To improve the estimation performance of the state estimator when there is a large linearization error in the system and to enhance the robustness of the estimator, we add penalties for sensitivity to the linearization error by adding the corresponding differential terms in Equation (28). The basic principle is that the deviation of the innovation process from its nominal value reflects the contribution of the linearization error to the prediction error of the Kalman filter concerning the equipment output. These deviations usually have complex expressions, making it difficult to mathematically handle the corresponding estimation problem. To streamline processing, we conduct a first-order approximation of the deviations via linearization around the origin. In Equation (29), $\mu_{t}\in \left(0,1\right]$ is a design parameter reflecting a trade-off between nominal estimation accuracy and penalization on the first-order approximation of deviations of the innovation process. Furthermore, $\psi_{t+1}$ is explicitly utilized and is generally obtainable in communications following the receipt of $y_{t+1}$. To enable access to this information, the only requirement is the incorporation of an indication code or timestamp into the communication channel. In the event that the measurement is successfully conveyed, as denoted by $\psi_{t+1}=1$, this cost function signifies that given the arrival of new information concerning $x_{t}$ at time instant t+1, its estimate should be updated in a robust fashion. If the remote robust state estimator is unable to receive the sensor measurement data packets, as denoted by $\psi_{t+1}=0$, then $y_{t+1}$ will be devoid of any information pertaining to the plant output. A recursive robust estimation algorithm can be derived through the application of this construction procedure.

**Theorem 2.**  *Assume that both $P_{t|t}$ and $Q_{t}$ are invertible, and $\left(1-\mu_{t}\right)/\mu_{t}$ is defined as $\lambda_{t}$. The state vector $x_{t+1}$ of the nonlinear system $\Sigma_{1}$ can be estimated based on $y_{k}{|}_{k=0}^{t+1}$ and Equations (28) and (29) using the following recursive process.*

*(1)*  *Linearization.*
(30) At(x^t∣t,εt1)=∂f(x,u)∂x|x=x^t∣t+εt1u=ut,
(31) $$\begin{array}{cc} C_{t}\left({\hat{x}}_{t|t},\varepsilon_{t}^{1}\right) & =\frac{\partial h(x)}{\partial x}|{}_{x={\hat{x}}_{t|t}+\varepsilon_{t}^{1}}, \end{array}$$
(32) $$\begin{array}{cc} {\hat{x}}_{t+1|t} & =f\left({\hat{x}}_{t|t}\right), \end{array}$$
(33) $$\begin{array}{cc} C_{t+1}\left({\hat{x}}_{t+1|t},\varepsilon_{t+1}^{2}\right) & =\frac{\partial h(x)}{\partial x}|{}_{x={\hat{x}}_{t+1|t}+\varepsilon_{t+1}^{2}}, \end{array}$$
  *where we abbreviate $A_{t}\left({\hat{x}}_{t|t},0\right)$, $C_{t}\left({\hat{x}}_{t|t},0\right)$, and $C_{t+1}\left({\hat{x}}_{t+1|t},0\right)$ as $A_{t}$, $C_{t}$, and $C_{t+1}$, respectively.*
*(2)*  *Parameter modification.*
(34) $$\begin{array}{cc} {\tilde{A}}_{t}\left(\varepsilon_{t}^{1},\varepsilon_{t+1}^{2}\right) & =\left[\begin{array}{ccc} A_{t}\left(\varepsilon_{t}^{1}\right) & 0_{n\times (d-1)m} & 0_{n\times m}\\ C_{t}\left(\varepsilon_{t}^{1}\right)-C_{t+1}\left(\varepsilon_{t+1}^{2}\right)A_{t}\left(\varepsilon_{t}^{1}\right) & 0_{m\times (d-1)m} & 0_{m\times m}\\ 0_{(d-1)m\times n} & I_{(d-1)m} & 0_{(d-1)m\times m} \end{array}\right], \end{array}$$
(35) $$\begin{array}{cc} {\tilde{B}}_{t}\left(\varepsilon_{t+1}^{2}\right) & =\left[\begin{array}{c} I_{n\times n}\\ -C_{t+1}\left(\varepsilon_{t+1}^{2}\right)\\ 0_{(d-1)m\times n} \end{array}\right], \end{array}$$
(36) $$\begin{array}{cc} {\tilde{C}}_{t}\left(\varepsilon_{t}^{1}\right) & =\left[C_{t}\left(\varepsilon_{t}^{1}\right)\;\overset{d}{\overbrace{I_{m}\cdots I_{m}}}\right], \end{array}$$
(37) $$\begin{array}{cc} {\hat{P}}_{t|t} & ={\left(P_{t|t}^{-1}+\lambda_{t}\psi_{t}S_{t}^{T}S_{t}\right)}^{-1}, \end{array}$$
(38) $$\begin{array}{cc} {\hat{Q}}_{t} & ={\left[Q_{t}^{-1}+\lambda_{t}\psi_{t+1}T_{t}^{T}{\left(I+\lambda_{t}S_{t}P_{t|t}S_{t}^{T}\right)}^{-1}T_{t}\right]}^{-1}, \end{array}$$
(39) $$\begin{array}{cc} {\hat{A}}_{t} & =\left[{\tilde{A}}_{t}-\lambda_{t}\psi_{t+1}{\hat{B}}_{t}{\hat{Q}}_{t}T_{t}^{T}S_{t}\right]\left[I-\lambda_{t}c_{t+1}\gamma_{t+1}{\hat{P}}_{t|t}S_{t}^{T}S_{t}\right], \end{array}$$
(40) $$\begin{array}{cc} {\hat{B}}_{1t} & ={\tilde{B}}_{t}-\lambda_{t}\psi_{t+1}\left({\hat{A}}_{t}P_{t|t}S_{t}^{T}+{\hat{B}}_{2t}{\hat{Q}}_{t}T_{t}^{T}\right)T_{t}, \end{array}$$
(41) $$\begin{array}{cc} {\hat{B}}_{2t} & ={\tilde{B}}_{t}-\lambda_{t}\psi_{t+1}{\tilde{A}}_{t}{\hat{P}}_{t|t}S_{t}^{T}T_{t}, \end{array}$$
(42) $$\begin{array}{cc} a_{t} & =f\left({\hat{x}}_{t|t},u_{t}\right)-A_{t}{\hat{x}}_{t|t}, \end{array}$$
(43) $$\begin{array}{cc} b_{t} & =h\left({\hat{x}}_{t|t-1}\right)-C_{t}{\hat{x}}_{t|t-1}, \end{array}$$
(44) $$\begin{array}{cc} S_{t}\left(\varepsilon_{t}^{1},\varepsilon_{t+1}^{2}\right) & =\left.\mathrm{col}\left\{{\left[\begin{array}{c} {\tilde{C}}_{t+1}\left(\varepsilon_{t+1}^{2}\right)\frac{\partial \left({\tilde{A}}_{t}\left(\varepsilon_{t}^{1},\varepsilon_{t+1}^{2}\right)\right)}{\partial \varepsilon_{t,j}^{1}}\\ \frac{\partial \left({\tilde{C}}_{t+1}\left(\varepsilon_{t+1}^{2}\right)\right)}{\partial \varepsilon_{t+1,j}^{2}}\frac{\partial \left({\tilde{A}}_{t}\left(\varepsilon_{t}^{1},\varepsilon_{t+1}^{2}\right)\right)}{\partial \varepsilon_{t+1,j}^{2}} \end{array}\right]}_{j=1}^{n}\right\}\right., \end{array}$$
(45) $$\begin{array}{cc} T_{t}\left(\varepsilon_{t}^{1},\varepsilon_{t+1}^{2}\right) & =\left.\mathrm{col}\left\{{\left[\begin{array}{c} \;\;\;\;0\\ \frac{\partial \left({\tilde{C}}_{t+1}\left(\varepsilon_{t+1}^{2}\right)\right)}{\partial \varepsilon_{t+1,j}^{2}}\frac{\partial \left({\tilde{B}}_{t+1}\left(\varepsilon_{t+1}^{2}\right)\right)}{\partial \varepsilon_{t+1,j}^{2}} \end{array}\right]}_{j=1}^{n}\right\}\right., \end{array}$$
(46) $$\begin{array}{cc} \Delta_{t} & =C_{t-1}\left(\varepsilon_{t-1}^{2}\right)x_{t-1}+b_{t-1}-C_{t}\left(\varepsilon_{t}^{2}\right)x_{t}-b_{t}, \end{array}$$
(47) $$\begin{array}{cc} D_{t} & =\left[\begin{array}{c} 0_{n\times m}\\ I_{m}\\ 0_{(d-1)m\times m} \end{array}\right], \end{array}$$
(48) $$\begin{array}{cc} d_{t} & =b_{t}-b_{t+1}, \end{array}$$
  *where we abbreviate $S_{t}\left(0,0,0\right)$, $T_{t}\left(0,0,0\right)$, ${\tilde{A}}_{t}\left(0,0,0\right)$, ${\tilde{B}}_{t}\left(0\right)$, and ${\tilde{C}}_{t}\left(0\right)$ as $S_{t}$, $T_{t}$, ${\tilde{A}}_{t}$, ${\tilde{B}}_{t}$, and ${\tilde{C}}_{t}$, respectively.*
*(3)*  *Time-update step: update of the state predictions and pseudo-covariance matrix of the prediction errors.*
(49) $$\begin{array}{cc} {\hat{X}}_{t+1|t} & ={\hat{A}}_{t}{\hat{X}}_{t|t}+D_{t}d_{t}+{\hat{B}}_{1t}a_{t}, \end{array}$$
(50) $$\begin{array}{cc} h\left({\hat{x}}_{t-d+1|t-d}\right) & ={\tilde{C}}_{t+1}{\hat{X}}_{t+1|t}+b_{t+1}, \end{array}$$
(51) $$\begin{array}{cc} P_{t+1|t} & =A_{t}{\hat{P}}_{t|t}A_{t}^{T}+{\hat{B}}_{2t}{\hat{Q}}_{t}{\hat{B}}_{2t}^{T}, \end{array}$$
(52) $$\begin{array}{cc} R_{e,t+1}^{} & =\psi_{t+1}{\tilde{C}}_{t+1}P_{t+1|t}{\tilde{C}}_{t+1}^{T}+R_{t+1}^{-1}. \end{array}$$
*(4)*  *Measurement-update step: update of the state estimation, pseudo-covariance matrix, and estimator gain.*
(53) $$\begin{array}{cc} K_{t+1} & ={\left[P_{t+1|t}^{-1}+\psi_{t+1}C_{t+1}^{T}R_{t+1}^{-1}C_{t+1}\right]}^{-1}C_{t+1}^{T}R_{t+1}^{-1}=P_{t+1|t+1}^{-1}C_{t+1}^{T}R_{t+1}^{-1}\\ \; & =P_{t+1|t}^{}C_{t+1}^{T}R_{e,t+1}^{-1}=P_{t+1|t}^{}C_{t+1}^{T}{\left(\psi_{t+1}C_{t+1}P_{t+1|t}C_{t+1}^{T}+R_{t+1}^{}\right)}^{-1}, \end{array}$$
(54) $$\begin{array}{cc} {\hat{X}}_{t+1|t+1} & ={\hat{X}}_{t+1|t}+\psi_{t+1}K_{t+1}\left(y_{t+1}-h\left({\hat{x}}_{t-d+1|t}\right)\right)\\ \; & ={\hat{A}}_{t}{\hat{X}}_{t|t}+D_{t}d_{t}+{\hat{B}}_{1t}a_{t}+\psi_{t+1}{\left[P_{t+1|t}^{-1}+\psi_{t+1}{\tilde{C}}_{t+1}^{T}R_{t+1}^{-1}{\tilde{C}}_{t+1}\right]}^{-1}\\ \; & \times {\tilde{C}}_{t+1}^{T}R_{t+1}^{-1}\left\{y_{t+1}-b_{t+1}-{\tilde{C}}_{t+1}\left({\hat{A}}_{t}{\hat{X}}_{t|t}+D_{t}d_{t}+{\hat{B}}_{1t}a_{t}\right)\right\}, \end{array}$$
(55) $$\begin{array}{cc} P_{t+1|t+1} & ={\left\{P_{t+1|t}^{-1}+\psi_{t+1}{\tilde{C}}_{t+1}^{T}R_{t+1}^{-1}{\tilde{C}}_{t+1}\right\}}^{-1}\\ \; & =\left(I-\psi_{t+1}K_{t+1}{\tilde{C}}_{t+1}\right)P_{t+1|t}\\ \; & =P_{t+1|t}-\psi_{t+1}P_{t+1|t}{\tilde{C}}_{t+1}^{T}{\left(\psi_{t+1}{\tilde{C}}_{t+1}P_{t+1|t}{\tilde{C}}_{t+1}^{T}+R_{t+1}^{}\right)}^{-1}{\tilde{C}}_{t+1}P_{t+1|t}, \end{array}$$
(56) $$\begin{array}{cc} {\hat{x}}_{t+1|t+1} & ={\left[{\hat{X}}_{t+1|t+1,1},{\hat{X}}_{t+1|t+1,2},\cdots ,{\hat{X}}_{t+1|t+1,n}\right]}^{T}, \end{array}$$
  *where ${\hat{X}}_{t|t,j}$ denotes the j-th component of vector ${\hat{X}}_{t|t}$.*

To demonstrate the theoretical results presented in this paper, the following well-known results from matrix analysis and linear estimations, as described in [33], are required.

**Lemma 1.**  *Assuming arbitrary matrices A, B, C, and D with dimensions that are compatible, it is postulated that all matrix inverses necessary for the calculations are present. Then,*

(57a) $$\begin{array}{cc} \left[\begin{array}{cc} A & B\\ C & D \end{array}\right] & =\left[\begin{array}{cc} I & 0\\ CA^{-1} & I \end{array}\right]\left[\begin{array}{cc} A & 0\\ 0 & D-CA^{-1}B \end{array}\right]\left[\begin{array}{cc} I & A^{-1}B\\ 0 & I \end{array}\right]\\ \; & =\left[\begin{array}{cc} I & BD^{-1}\\ 0 & I \end{array}\right]\left[\begin{array}{cc} A-BD^{-1}C & 0\\ 0 & D \end{array}\right]\left[\begin{array}{cc} I & 0\\ D^{-1}C & I \end{array}\right] \end{array}$$

(57b) $$\begin{array}{cc} {\left[A+BCD\right]}^{-1} & =A^{-1}-A^{-1}B{\left[C^{-1}+DA^{-1}B\right]}^{-1}DA^{-1} \end{array}$$

(57c) $$\begin{array}{cc} A{\left(I+BA\right)}^{-1} & ={\left(I+AB\right)}^{-1}A. \end{array}$$

**Proof of Theorem 2.**  In the interest of brevity, we define the vectors $\alpha_{t0}$ and $\alpha_{t}$ as $\alpha_{t0}=col\left\{{\hat{X}}_{t|t},0\right\}$ and $\alpha_{t}=col\left\{X_{t|t+1},w_{t|t+1}\right\}$, respectively. Furthermore, we define the matrices ${\hat{P}}_{t|t}$, ${\hat{Q}}_{t}$, ${\hat{B}}_{1t}$, ${\hat{B}}_{2t}$, and ${\hat{A}}_{t}$ as (37)–(41), respectively.

It should be noted that for all k∈{1,2,…,n}, the following is satisfied:

(58) $$\begin{array}{cc} \frac{\partial e_{t}\left(\varepsilon_{t}^{1}\varepsilon_{t+1}^{2}\right)}{\partial \varepsilon_{t,k}^{1}} & ={\tilde{C}}_{t+1}\left(\varepsilon_{t+1}^{2}\right)\frac{\partial {\tilde{A}}_{t}\left(\varepsilon_{t}^{1},\varepsilon_{t+1}^{2}\right)}{\partial \varepsilon_{t,k}^{1}}X_{t|t+1}, \end{array}$$

(59) $$\begin{array}{cc} \frac{\partial e_{t}\left(\varepsilon_{t}^{1},\varepsilon_{t+1}^{2}\right)}{\partial \varepsilon_{t+1,k}^{2}} & =\frac{\partial {\tilde{C}}_{t+1}\left(\varepsilon_{t+1}^{2}\right)}{\partial \varepsilon_{t+1,k}^{2}}\left[\frac{\partial {\tilde{A}}_{t}\left(\varepsilon_{t}^{1},\varepsilon_{t+1}^{2}\right)}{\partial \varepsilon_{t+1,k}^{2}}X_{t|t+1}+\frac{\partial {\tilde{B}}_{t+1}\left(\varepsilon_{t+1}^{2}\right)}{\partial \varepsilon_{t+1,k}^{2}}a_{t}+\frac{\partial {\tilde{B}}_{t+1}\left(\varepsilon_{t+1}^{2}\right)}{\partial \varepsilon_{t+1,k}^{2}}w_{t|t+1}\right]. \end{array}$$

It can be easily shown, based on the definition of the cost function $J\left(X_{t|t+1},w_{t|t+1}\right)$, that

(60) $$\begin{array}{cc} \; & J\left(\alpha_{t}\right)=\frac{\mu_{t}}{2}\left\{{\left(*\right)}^{T}diag\left\{P_{t|t}^{-1},Q_{t}^{-1}\right\}\left(\alpha_{t}-\alpha_{t0}\right)+\psi_{t+1}{\left(*\right)}^{T}R_{t+1}^{-1}\right.\\ \; & \left.\times \left[b_{t+1}+{\tilde{C}}_{t+1}\left(\left[{\tilde{A}}_{t}\;{\tilde{B}}_{t}\right]\alpha_{t}+D_{t}d_{t}+{\tilde{B}}_{t}a_{t}\right)-y_{t+1}\right]+\lambda_{t}\psi_{t+1}{\left(*\right)}^{T}\left(\left[S_{t}\;T_{t}\right]\alpha_{t}+T_{t}a_{t}\right)\right\}. \end{array}$$

Therefore.

(61) $$\begin{array}{cc} \frac{\partial J\left(\alpha_{t}\right)}{\partial \alpha_{t}}=\mu_{t} & \left\{diag\left\{P_{t|t}^{-1},Q_{t}^{-1}\right\}\left(\alpha_{t}-\alpha_{t0}\right)+\psi_{t+1}\right.{\left({\tilde{C}}_{t+1}\left[{\tilde{A}}_{t}\;{\tilde{B}}_{t}\right]\right)}^{T}\\ \; & \times R_{t+1}^{-1}\left[b_{t+1}+{\tilde{C}}_{t+1}\left(\left[{\tilde{A}}_{t}\;{\tilde{B}}_{t}\right]\alpha_{t}+D_{t}d_{t}+{\tilde{B}}_{t}a_{t}\right)-y_{t+1}\right]\\ \; & \left.+\lambda_{t}\psi_{t+1}{\left[S_{t}\;T_{t}\right]}^{T}\left(\left[S_{t}\;T_{t}\right]\alpha_{t}+T_{t}a_{t}\right)\right\}\\ =\mu_{t} & \left\{\left(diag\left\{P_{t|t}^{-1},Q_{t}^{-1}\right\}+\lambda_{t}\psi_{t+1}{\left[S_{t}\;T_{t}\right]}^{T}\left[S_{t}\;T_{t}\right]\right.+\psi_{t+1}{\left[{\tilde{A}}_{t}\;{\tilde{B}}_{t}\right]}^{T}{{\tilde{C}}_{t+1}}^{T}R_{t+1}^{-1}{\tilde{C}}_{t+1}\left[{\tilde{A}}_{t}\;{\tilde{B}}_{t}\right]\right)\alpha_{t}\\ \; & -diag\left\{P_{t|t}^{-1},Q_{t}^{-1}\right\}\alpha_{t0}+\psi_{t+1}{\left[{\tilde{A}}_{t}\;{\tilde{B}}_{t}\right]}^{T}{{\tilde{C}}_{t+1}}^{T}R_{t+1}^{-1}\left(b_{t+1}-y_{t+1}\right)\\ \; & +\psi_{t+1}\left({\left[{\tilde{A}}_{t}\;{\tilde{B}}_{t}\right]}^{T}{{\tilde{C}}_{t+1}}^{T}R_{t+1}^{-1}{\tilde{C}}_{t+1}{\tilde{B}}_{t}+\lambda_{t}{\left[S_{t}\;T_{t}\right]}^{T}T_{t}\right)a_{t}\\ \; & \left.+\psi_{t+1}{\left[{\tilde{A}}_{t}\;{\tilde{B}}_{t}\right]}^{T}{{\tilde{C}}_{t+1}}^{T}R_{t+1}^{-1}{\tilde{C}}_{t+1}D_{t}d_{t}\right\}. \end{array}$$

It should be noted that $J\left(\alpha_{t}\right)$ is a convex function and $\mu_{t}\neq 0$. The optimal value of $\alpha_{t}$, denoted as ${\hat{\alpha }}_{t}$, which minimizes $J\left(\alpha_{t}\right)$, is determined by its first-order derivative condition. That is,

(62) $$\begin{array}{cc} {\hat{\alpha }}_{t}= & {\left(diag\left\{P_{t|t}^{-1},Q_{t}^{-1}\right\}+\lambda_{t}\psi_{t+1}{\left[S_{t}\;T_{t}\right]}^{T}\left[S_{t}\;T_{t}\right]+\psi_{t+1}{\left[{\tilde{A}}_{t}\;{\tilde{B}}_{t}\right]}^{T}{{\tilde{C}}_{t+1}}^{T}R_{t+1}^{-1}{\tilde{C}}_{t+1}\left[{\tilde{A}}_{t}\;{\tilde{B}}_{t}\right]\right)}^{-1}\\ \; & \times \left\{diag\left\{P_{t|t}^{-1},Q_{t}^{-1}\right\}\alpha_{t0}-\psi_{t+1}{\left[{\tilde{A}}_{t}\;{\tilde{B}}_{t}\right]}^{T}{{\tilde{C}}_{t+1}}^{T}R_{t+1}^{-1}\left(b_{t+1}-y_{t+1}\right)\right.\\ \; & \left.-\psi_{t+1}\left({\left[{\tilde{A}}_{t}\;{\tilde{B}}_{t}\right]}^{T}{{\tilde{C}}_{t+1}}^{T}R_{t+1}^{-1}{\tilde{C}}_{t+1}{\tilde{B}}_{t}+\lambda_{t}{\left[S_{t}\;T_{t}\right]}^{T}T_{t}\right)a_{t}-\psi_{t+1}{\left[{\tilde{A}}_{t}\;{\tilde{B}}_{t}\right]}^{T}{{\tilde{C}}_{t+1}}^{T}R_{t+1}^{-1}{\tilde{C}}_{t+1}D_{t}d_{t}\right\}. \end{array}$$

We can directly utilize the inverse application of the matrix inversion in Lemma (57b) to obtain the following equation:

(63) $$\begin{array}{c} T_{t}^{T}T_{t}-\lambda_{t}\psi_{t+1}T_{t}^{T}S_{t}{\left[P_{t|t}^{-1}+\lambda_{t}\psi_{t+1}S_{t}^{T}S_{t}\right]}^{-1}S_{t}^{T}T_{t}=T_{t}^{T}{\left[I+\lambda_{t}\psi_{t+1}S_{t}P_{t|t}S_{t}^{T}\right]}^{-1}T_{t} \end{array}.$$

Then, from Lemma 1 and the definitions of the matrices $P_{t|t}^{-1}$ and $Q_{t}^{-1}$, we can immediately obtain the following relation:

(64) $$\begin{array}{cc} \; & diag\left\{P_{t|t}^{-1},Q_{t}^{-1}\right\}+\lambda_{t}\psi_{t+1}{\left[S_{t}T_{t}\right]}^{T}\left[S_{t}T_{t}\right]\\ \; & =\left[\begin{array}{cc} I & 0\\ \lambda_{t}\psi_{t+1}T_{t}^{T}S_{t}{\hat{P}}_{t|t} & I \end{array}\right]\left[\begin{array}{cc} {\hat{P}}_{t|t}^{-1} & 0\\ 0 & {\hat{Q}}_{t}^{-1} \end{array}\right]\left[\begin{array}{cc} I & \lambda_{t}\psi_{t+1}{\hat{P}}_{t|t}S_{t}^{T}T_{t}\\ 0 & I \end{array}\right]. \end{array}$$

By substituting this relation into (62), it can be further proved that

(65) $$\begin{array}{cc} {\hat{\alpha }}_{t}= & \left[\begin{array}{cc} I & \;\;-\lambda_{t}\psi_{t+1}{\hat{P}}_{t|t}S_{t}^{T}T_{t}\\ 0 & \;\;I \end{array}\right]{\left\{\left[\begin{array}{cc} {\hat{P}}_{t|t}^{-1} & 0\\ 0 & {\hat{Q}}_{t}^{-1} \end{array}\right]+\psi_{t+1}{\left[{\tilde{A}}_{t}\;{\hat{B}}_{2t}\right]}^{T}{\tilde{C}}_{t+1}^{T}R_{t+1}^{-1}{\tilde{C}}_{t+1}\left[{\tilde{A}}_{t}\;{\hat{B}}_{2t}\right]\right\}}^{-1}\\ \; & \times \left\{\mathrm{col}\left\{I,-\lambda_{t}\psi_{t+1}T_{t}^{T}S_{t}{\hat{P}}_{t|t}\right\}P_{t|t}^{-1}{\hat{X}}_{t|t}+\psi_{t+1}{\left[{\tilde{A}}_{t}\;{\hat{B}}_{2t}\right]}^{T}{\tilde{C}}_{t+1}^{T}R_{t+1}^{-1}\left(y_{t+1}-b_{t+1}\right)\right.\\ \; & \left.-\psi_{t+1}\left({\left[{\tilde{A}}_{t}\;{\hat{B}}_{2t}\right]}^{T}{\tilde{C}}_{t+1}^{T}R_{t+1}^{-1}{\tilde{C}}_{t+1}{\tilde{B}}_{t}+\lambda_{t}\left[\begin{array}{cc} I & \;\;0\\ -\lambda_{t}\psi_{t+1}T_{t}^{T}S_{t}{\hat{P}}_{t|t} & \;\;I \end{array}\right]{\left[S_{t}\;T_{t}\right]}^{T}T_{t}\right)a_{t}\right.\\ \; & \left.-\psi_{t+1}{\left[{\tilde{A}}_{t}\;{\hat{B}}_{2t}\right]}^{T}{{\tilde{C}}_{t+1}}^{T}R_{t+1}^{-1}{\tilde{C}}_{t+1}D_{t}d_{t}\right\}. \end{array}$$

Hence,

(66) $$\begin{array}{cc} \; & {\hat{X}}_{t+1|t+1}=\left[{\tilde{A}}_{t}\;{\tilde{B}}_{t}\right]\alpha_{t}+D_{t}d_{t}+{\tilde{B}}_{t}a_{t}\\ = & \left[{\tilde{A}}_{t}\;{\hat{B}}_{2t}\right]{\left\{I+\psi_{t+1}diag\left\{{\hat{P}}_{t|t},{\hat{Q}}_{t}\right\}{\left[{\tilde{A}}_{t}\;{\hat{B}}_{2t}\right]}^{T}{\tilde{C}}_{t+1}^{T}R_{t+1}^{-1}{\tilde{C}}_{t+1}\left[{\tilde{A}}_{t}\;{\hat{B}}_{2t}\right]\right\}}^{-1}diag\left\{{\hat{P}}_{t|t},{\hat{Q}}_{t}\right\}\\ \times & \left\{\mathrm{col}\left\{I,-\lambda_{t}\psi_{t+1}T_{t}^{T}S_{t}{\hat{P}}_{t|t}\right\}P_{t|t}^{-1}{\hat{X}}_{t|t}+\psi_{t+1}{\left[{\tilde{A}}_{t}\;{\hat{B}}_{2t}\right]}^{T}{\tilde{C}}_{t+1}^{T}R_{t+1}^{-1}\left(y_{t+1}-b_{t+1}\right)\right.\\ \; & \left.-\psi_{t+1}\left({\left[{\tilde{A}}_{t}\;{\hat{B}}_{2t}\right]}^{T}{\tilde{C}}_{t+1}^{T}R_{t+1}^{-1}{\tilde{C}}_{t+1}{\tilde{B}}_{t}+\lambda_{t}\left[\begin{array}{cc} I & \;\;0\\ -\lambda_{t}\psi_{t+1}T_{t}^{T}S_{t}{\hat{P}}_{t|t} & \;\;I \end{array}\right]{\left[S_{t}\;T_{t}\right]}^{T}T_{t}\right)a_{t}\right.\\ \; & \left.-\psi_{t+1}{\left[{\tilde{A}}_{t}\;{\hat{B}}_{2t}\right]}^{T}{{\tilde{C}}_{t+1}}^{T}R_{t+1}^{-1}{\tilde{C}}_{t+1}D_{t}d_{t}\right\}+D_{t}d_{t}+{\tilde{B}}_{t}a_{t}\\ = & {\left[I+\psi_{t+1}P_{t+1|t}{\tilde{C}}_{t+1}^{T}R_{t+1}^{-1}{\tilde{C}}_{t+1}\right]}^{-1}\left\{{\hat{A}}_{t}{\hat{X}}_{t|t}+\psi_{t+1}P_{t+1|t}{\tilde{C}}_{t+1}^{T}R_{t+1}^{-1}\left(y_{t+1}-b_{t+1}\right)\right.\\ \; & \left.-\psi_{t+1}\left(P_{t+1|t}{\tilde{C}}_{t+1}^{T}R_{t+1}^{-1}{\tilde{C}}_{t+1}{\tilde{B}}_{t}+\lambda_{t}\left({\hat{A}}_{t}P_{t|t}S_{t}^{T}+{\hat{B}}_{2t}{\hat{Q}}_{t}T_{t}^{T}\right)T_{t}\right)a_{t}-\psi_{t+1}P_{t+1|t}{{\tilde{C}}_{t+1}}^{T}R_{t+1}^{-1}{\tilde{C}}_{t+1}D_{t}d_{t}\right\}\\ \; & +D_{t}d_{t}+{\tilde{B}}_{t}a_{t}, \end{array}$$

where $P_{t+1|t}=\left[{\tilde{A}}_{t}\;{\hat{B}}_{2t}\right]diag\left\{{\hat{P}}_{t|t},{\hat{Q}}_{t}\right\}{\left[{\tilde{A}}_{t}\;{\hat{B}}_{2t}\right]}^{T}={\tilde{A}}_{t}{\hat{P}}_{t|t}{\tilde{A}}_{t}^{T}+{\hat{B}}_{2t}{\hat{Q}}_{t}{\hat{B}}_{2t}^{T}$.

In the derivation of the above equation, the relation ${\hat{P}}_{t|t}P_{t|t}^{-1}=I-\lambda_{t}\psi_{t+1}{\hat{P}}_{t|t}S_{t}^{T}S_{t}$ is utilized, which is a direct result of the definition of the matrix. By adding the relation ${\left[I+\psi_{t+1}P_{t+1|t}C_{t+1}^{T}R_{t+1}^{-1}C_{t+1}\right]}^{-1}=I-\psi_{t+1}{\left[I+\psi_{t+1}P_{t+1|t}C_{t+1}^{T}R_{t+1}^{-1}C_{t+1}\right]}^{-1}P_{t+1|t}C_{t+1}^{T}R_{t+1}^{-1}C_{t+1}$ to the above equation, we can finally obtain

(67) $$\begin{array}{cc} {\hat{X}}_{t+1|t+1}= & {\hat{A}}_{t}{\hat{X}}_{t|t}-\psi_{t+1}{\left[I+\psi_{t+1}P_{t+1|t}{\tilde{C}}_{t+1}^{T}R_{t+1}^{-1}{\tilde{C}}_{t+1}\right]}^{-1}P_{t+1|t}{\tilde{C}}_{t+1}^{T}R_{t+1}^{-1}{\tilde{C}}_{t+1}{\hat{A}}_{t}{\hat{X}}_{t|t}\\ \; & +\psi_{t+1}{\left[I+\psi_{t+1}P_{t+1|t}{\tilde{C}}_{t+1}^{T}R_{t+1}^{-1}{\tilde{C}}_{t+1}\right]}^{-1}P_{t+1|t}{\tilde{C}}_{t+1}^{T}R_{t+1}^{-1}\left(y_{t+1}-b_{t+1}\right)\\ \; & -\psi_{t+1}{\left[I+\psi_{t+1}P_{t+1|t}{\tilde{C}}_{t+1}^{T}R_{t+1}^{-1}{\tilde{C}}_{t+1}\right]}^{-1}P_{t+1|t}{\tilde{C}}_{t+1}^{T}R_{t+1}^{-1}{\tilde{C}}_{t+1}{\tilde{B}}_{t}a_{t}\\ \; & -\psi_{t+1}\lambda_{t}\left({\hat{A}}_{t}P_{t|t}S_{t}^{T}+{\hat{B}}_{2t}{\hat{Q}}_{t}T_{t}^{T}\right)T_{t}a_{t}+{\tilde{B}}_{t}a_{t}\\ \; & +\psi_{t+1}^{2}\lambda_{t}{\left[I+\psi_{t+1}P_{t+1|t}{\tilde{C}}_{t+1}^{T}R_{t+1}^{-1}{\tilde{C}}_{t+1}\right]}^{-1}P_{t+1|t}{\tilde{C}}_{t+1}^{T}R_{t+1}^{-1}{\tilde{C}}_{t+1}\\ \; & \times \left({\hat{A}}_{t}P_{t|t}S_{t}^{T}+{\hat{B}}_{2t}{\hat{Q}}_{t}T_{t}^{T}\right)T_{t}a_{t}\\ \; & -\psi_{t+1}{\left[I+\psi_{t+1}P_{t+1|t}{\tilde{C}}_{t+1}^{T}R_{t+1}^{-1}{\tilde{C}}_{t+1}\right]}^{-1}P_{t+1|t}{{\tilde{C}}_{t+1}}^{T}R_{t+1}^{-1}{\tilde{C}}_{t+1}D_{t}d_{t}+D_{t}d_{t}\\ = & {\hat{A}}_{t}{\hat{X}}_{t|t}+D_{t}d_{t}+{\hat{B}}_{1t}a_{t}+\psi_{t+1}{\left[P_{t+1|t}^{-1}+\psi_{t+1}{\tilde{C}}_{t+1}^{T}R_{t+1}^{-1}{\tilde{C}}_{t+1}\right]}^{-1}\\ \; & \times {\tilde{C}}_{t+1}^{T}R_{t+1}^{-1}\left\{y_{t+1}-b_{t+1}-{\tilde{C}}_{t+1}\left({\hat{A}}_{t}{\hat{X}}_{t|t}+D_{t}d_{t}+{\hat{B}}_{1t}a_{t}\right)\right\}. \end{array}$$

The proof of Theorem 2 ends here. □

## 4. Numerical Simulations

**Figure 5:**
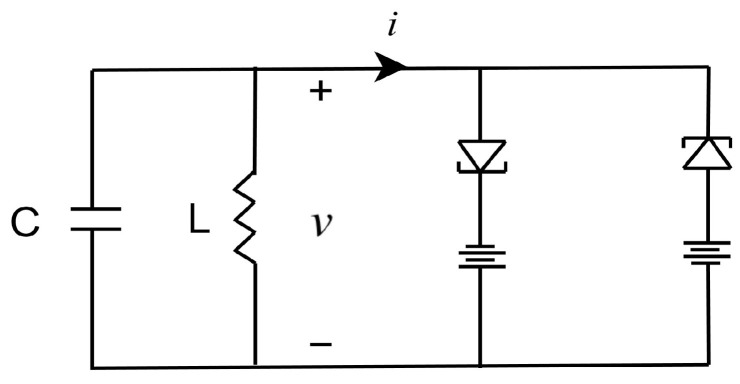
Oscillation circuit consisting of a double-tunnel diode negative-resistance circuit.

In this section, we choose a simple yet representative nonlinear system as the object observed by the sensor since its highly representative nonlinear characteristics can well reflect the superiority of the robust state estimation algorithm designed in this paper. As illustrated in Figure 5, the voltage value *v* of the oscillation circuit consisting of a double-tunnel diode negative-resistance circuit, as described in [34], is selected as the estimation target. When the negative-resistance circuit satisfies characteristic $i=-v+\frac{1}{3}v^{3}$, the circuit equation can be rewritten as the following equation:

$$\ddot{v}-\varsigma \left(1-v^{2}\right)\dot{v}+v=0,$$

as described in [35], which is known as the Van der Pol equation. Within the above equation, $\varsigma =\sqrt{\frac{L}{C}}$, $\dot{v}=\frac{dv}{d\iota }=\sqrt{CL}\frac{dv}{dt}$, $\ddot{v}=\frac{d^{2}v}{d\iota^{2}}=CL\frac{d^{2}v}{dt^{2}}$, where $\iota =\frac{1}{\sqrt{CL}}$. By using the Van der Pol oscillator as a simplified representation, we can concentrate on the fundamental principles and methods of our proposed algorithm, which can then be applied to more complex actual nonlinear systems.

Consider a sensor that monitors the voltage of the Van der Pol oscillator and sends its measurements to an event trigger. The event trigger determines whether to send the data to a remote robust state estimator through a wireless communication network. We take into account the effects of transmission delays and DoS attacks during communication. The entire process is shown in Figure 1. We choose the state vector $x={\left[x_{1},x_{2}\right]}^{T}={\left[v,\dot{v}\right]}^{T}$, the measurement vector $y=x_{1}=v$, and the system parameter ς=0.6. The state equation and the measurement equation are shown below:

$$\begin{array}{cc} \; & {\dot{x}}_{1}=x_{2},\\ \; & {\dot{x}}_{2}=\varsigma \left(1-x_{1}^{2}\right)x_{2}-x_{1},\varsigma =0.6,\\ \; & y=x_{1}. \end{array}$$

By employing the forward Euler method with a sampling interval of T=0.2 to discretize the above state-space equation and introducing the process noise $w_{t}$, the measurement noise $\vartheta_{t}$, the packet-arrival parameter $\psi_{t}$ and the number of measurement delays *d*, we derive the following discrete state-space model:

$$\begin{array}{c} x_{t+1}=f\left(x_{t}\right)+\omega_{t}=\left[\begin{array}{c} x_{t,1}+Tx_{t,2}\\ x_{t,2}+\varsigma T\left(1-x_{t,1}^{2}\right)x_{t,2}-Tx_{t,1} \end{array}\right]+\omega_{t},\\ y_{t}=\psi_{t}\left[1,0\right]x_{t-d}+\vartheta_{t}. \end{array}$$

By expanding the state equation using first-order Taylor linearization at ${\hat{x}}_{t|t}$, ignoring higher-order terms, and introducing error parameters $\varepsilon_{t}$, we can obtain

$$\begin{array}{c} x_{t+1}=A_{t}x_{t}\left(\varepsilon_{t}^{1}\right)+a_{t}+w_{t},\\ y_{t}=\psi_{t}\left[1,0\right]x_{t-d}+\vartheta_{t}, \end{array}$$

where $A_{t}=\frac{\partial f(x)}{\partial x}|{}_{x={\hat{x}}_{t|t}}$ and $a_{t}=f\left({\hat{x}}_{t|t}\right)-A_{t}{\hat{x}}_{t|t}$. We assign a value of $10^{-2}$ to the covariance of the process noise and a value of 1 to the covariance of the measurement noise. The initial pseudo-covariance matrix of the state is set to $P_{0}=I_{2}$, and the initial state is set to $x_{0}={\left[0.5,0\right]}^{T}$. We set the number of measurement delays d = 2, which means that there is a delay of two sampling intervals from when the measurement value is sent from the event trigger to when it is received by the remote robust state estimator. More precisely, when utilizing timestamp technology, the number of measurement-delay frames is 2.

The variance of the ensemble-average estimation errors at each sampling instant is calculated through $6\times 10^{2}$ random numerical simulations. The temporal variable, *t*, is varied from 0 to $2\times 10^{2}$ in the numerical simulations conducted. By evaluating the estimation error using the Euclidean distance between the actual and predicted values, the performance of the two estimation algorithms is compared. The variance of the ensemble-average estimation errors for these $6\times 10^{2}$ random simulations at each sampling instant is computed as follows:

$$\begin{array}{c} E∥x_{t}-{\hat{x}}_{t|t}|{|}^{2}\approx \frac{1}{600}\sum_{j=1}^{600}∥x_{t}-{{\hat{x}}^{j}}_{t|t}|{|}^{2}, \end{array}$$

where *j* represents the serial number of the random simulations. The root mean square error of the *k*-th state component at each time instant is calculated as $\sqrt{\frac{1}{600}\sum_{j=1}^{600}{\left(x_{t,k}-{{\hat{x}}^{j}}_{t|t,k}\right)}^{2}}$.

**Figure 6:**
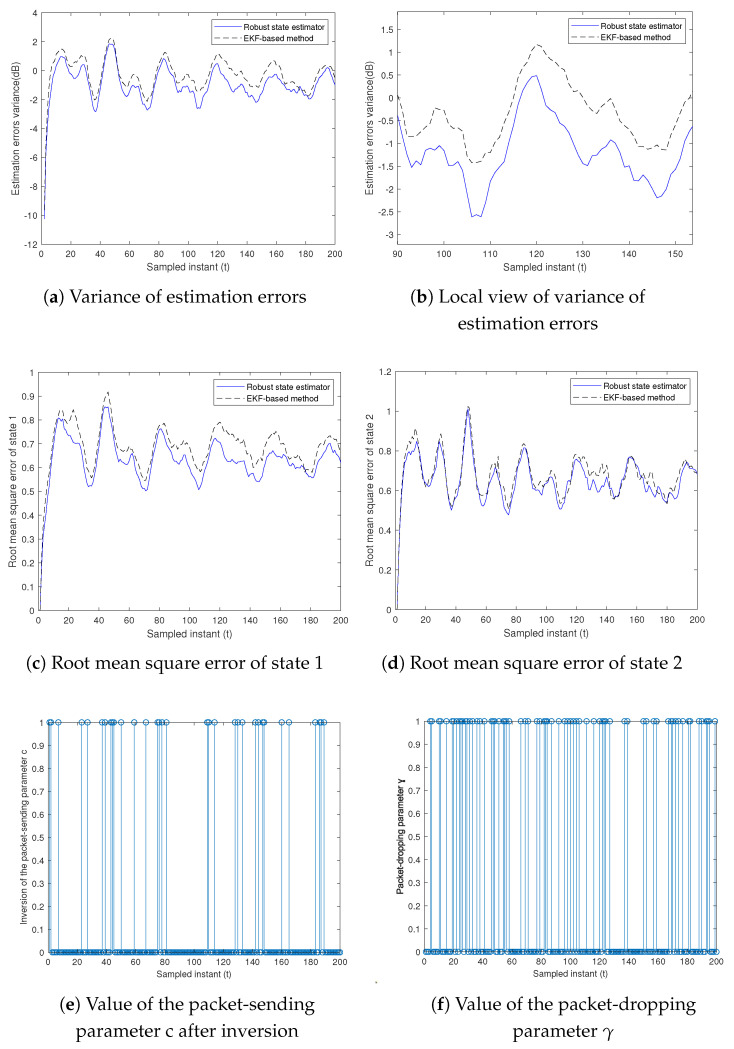
State estimation with packet transmission rate α=0.85 and packet-dropping rate ρ=0.6.

The measurement-dropping parameter $\gamma_{t}$ is represented by a stationary Bernoulli process with an expected rate of ρ=0.6. We set the packet transmission rate to a value of 0.85. The design parameter $\mu_{t}$ is assigned a value of 0.86. In Figure 6a, the variance of the ensemble-average estimation errors with respect to the time variation for the two state estimation algorithms is demonstrated. In order to clarify the differences between the curves, they are partially re-plotted in Figure 6b. It is evident that the overall variance of the estimation errors for both the voltage and its derivative, as obtained by the robust state estimator based on sensitivity penalization, is significantly superior to that of the EKF-based method that utilizes nominal parameter values. This indicates that the remote robust state estimator based on sensitivity penalization can effectively handle linearization errors to some extent. The root mean square error of the states is also shown in Figure 6c,d. For a more intuitive presentation of the results, Figure 6e illustrates the inversion of the packet-sending parameter, with “0” representing packet transmission and “1” denoting no packet transmission. Figure 6f shows the resulting packet-dropping parameter when the transmission network experiences DoS attacks, where “0” indicates packet loss and “1” represents normal packet transmission. This aligns with the characteristics of DoS attacks, specifically that the attack duration is bounded and the packet loss changes over time due to the energy restrictions of the attacker. In Table 1, we quantitatively compare the variance of the estimation errors and the root mean square error for the two different algorithms. By calculating the averages of the variance of the estimation errors and root mean square error in Figure 6 for 200 moments and 600 experiments, we obtain the EEV and RMSE values listed in Table 1. As shown in Table 1, the robust state estimator has a lower variance of the estimation errors of 0.0984 compared to that of the EKF-based method, and the root mean square error of the two state components estimated by the robust state estimator is also lower than that of the EKF-based method. It is obvious that at the same packet-dropping rate, the performance of the robust state estimator designed in this paper is better than that of the EKF-based method.

**Figure 7:**
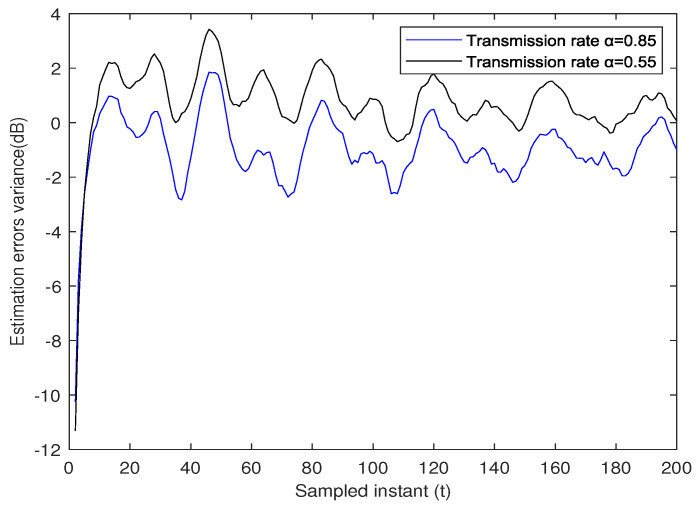
Variance of estimation errors with different transmission rates α.

The variance of the ensemble-average estimation errors of the robust state estimator under two different transmission rates, α=0.85 and α=0.55, is depicted in Figure 7. The analysis of the data reveals that higher transmission rates lead to improved estimation accuracy, although this comes with an associated increase in the transmission burden.

## 5. Conclusions

In this paper, the problem of event-triggered robust state estimation for nonlinear networked systems with a constant measurement delay against DoS attacks is investigated. We utilize a state augmentation method to transform the measurement-delay model into a formally non-delayed model. Two Bernoulli distributions are employed in the packet-sending and packet-loss processes, respectively, to explicitly indicate whether a packet is sent or lost. A robust state estimator is derived with an explicit packet-arrival parameter by penalizing the sensitivity of estimation errors with respect to linearization errors. For this new robust estimator, an analytical solution akin to the EKF is developed, enabling recursive implementation. Simulation results demonstrate that our proposed method offers appreciably improved estimation accuracy compared to the EKF-based approach. Although our robust state estimation algorithm can improve estimation performance, further studies are required to systematically determine the optimal value for the design parameter μ.

## Figures and Tables

**Table 1:** Comparison of the variance of the estimation errors and root mean square error.

	EEV	RMSE of $x_{1}$	RMSE of $x_{2}$
EKF-based method	0.9585	0.6891	0.6814
Robust state estimator	0.8601	0.6336	0.6556

## Data Availability

The data that support the findings of this study are available from the corresponding author upon reasonable request.
